# Pre-existing asthma as a comorbidity does not modify cytokine responses and severity of COVID-19

**DOI:** 10.1186/s13223-021-00569-8

**Published:** 2021-07-08

**Authors:** Jian Luo, Yi-Ling Chen, Wentao Chen, David A. Duncan, Alexander Mentzer, Julian C. Knight, Graham Ogg, Paul Klenerman, Ian D. Pavord, Luzheng Xue

**Affiliations:** 1 Respiratory Medicine Unit, and Oxford NIHR Biomedical Research Centre, University of Oxford, Oxford, UK; 2 MRC Human Immunology Unit, Weatherall Institute of Molecular Medicine, University of Oxford, Oxford, UK; 3 Diamond Light Source, Harwell Science and Innovation Campus, Didcot, UK; 4 Wellcome Centre for Human Genetics, University of Oxford, Oxford, UK; 5 Translational Gastroenterology Unit and Peter Medawar Building, University of Oxford, Oxford, UK; 6 Respiratory Medicine Unit, Nuffield Department of Medicine, John Radcliffe Hospital, University of Oxford, Oxford, UK

**Keywords:** Asthma, COVID-19, Cytokine storm, Mortality

## Abstract

**Background:**

A significant portion of COVID-19 sufferers have asthma. The impacts of asthma on COVID-19 progression are still unclear but a modifying effect is plausible as respiratory viruses are acknowledged to be an important trigger for asthma exacerbations and a different, potentially type-2 biased, immune response might occur. In this study, we compared the blood circulating cytokine response to COVID-19 infection in patients with and without asthma.

**Methods:**

Plasma samples and clinical information were collected from 80 patients with mild (25), severe (36) or critical (19) COVID-19 and 29 healthy subjects at the John Radcliffe Hospital, Oxford, UK. The concentrations of 51 circulating proteins in the plasma samples were measured with Luminex and compared between groups.

**Results:**

Total 16 pre-existing asthma patients were found (3 in mild, 10 in severe, and 3 in critical COVID-19). The prevalence of asthma in COVID-19 severity groups did not suggest a clear correlation between asthma and COVID-19 severity. Within the same COVID-19 severity group, no differences were observed between patients with or without asthma on oxygen saturation, CRP, neutrophil counts, and length of hospital stay. The mortality in the COVID-19 patients with asthma (12.5%) was not higher than that in patients without asthma (17.2%). No significant difference was found between asthmatic and non-asthmatic in circulating cytokine response in different COVID-19 severity groups, including the cytokines strongly implicated in COVID-19 such as CXCL10, IL-6, CCL2, and IL-8.

**Conclusions:**

Pre-existing asthma was not associated with an enhanced cytokine response after COVID-19 infection, disease severity or mortality.

## Background

Since the outbreak in Wuhan, China, COVID-19 has spread through the world at an unprecedented speed, causing more than 92 million infections and over 2 million deaths worldwide at the time of writing. With the increased studies and researches on COVID-19, it is clear that SARS-CoV-2, an enveloped, positive-sense single-stranded RNA virus [1], is the pathogen causing this global pandemic; it invades the host cells through angiotensin-converting enzyme 2 (ACE2) receptor [2, 3] and results in respiratory failure and a lethal state of acute respiratory distress syndrome (ARDS) by diffuse alveolar damage, pulmonary capillary congestion, and cytokine storm [4, 5].

Asthma is a common airway disease with more than 300 million patients worldwide [6]. Both COVID-19 and asthma are associated with pulmonary inflammation, damage and dysfunction, and can result in respiratory failure and death. However, the influence of asthma on the process and outcomes of COVID-19 is unclear. Respiratory viruses such as influenza virus and other coronaviruses are common causes for asthma exacerbations [7]. The possibility of a type-2 deviated immune response during viral infections has been speculated [8]. Th2-skewed immunity down-regulates type I and III interferon productions, which are the key contributors of late-phase hyperinflammation in severe respiratory viral diseases [9], and the deficiency in interferons is also linked to the impaired innate antiviral immune responses in asthma [10]. Existing data on asthma as a risk factor for COVID-19 is contradictory. Large cohort studies in different geographic regions found that the prevalence of asthma in COVID-19 was under-represented and lower than that in general populations [11–13]. A study of 961 hospitalized COVID-19 patients did not show an increase of severity and mortality in patients with asthma [14]. In addition, the Th2 cytokines and eosinophilic inflammation, hallmarks of asthma pathophysiology, have been demonstrated to be associated with decreased ACE2 expression in bronchial epithelium of asthmatic patients [14–17]. Hence, asthma is unlikely to represent a risk factor of COVID-19 infection [9]. Nevertheless, in vitro study found that Rhinovirus infection could increase the ACE2 expression in nasal tissues from patients with asthma [18]. Meanwhile, a recent study of a population of more than 17 million has reported that severe asthma is associated with a higher occurrence of COVID-19 related deaths [19]. Therefore, the relationships and interactions between asthma and COVID-19 are still unclear.

Cytokine “storm” has been recognised as an important driver of disease deterioration and severity in COVID-19 [18, 20]. However, there is still limited understanding of the impact of asthma on COVID-19-derived cytokine storm, with only one study reporting no difference between patients with and without asthma in the levels of interleukin (IL)-2, IL-6, IL-10, IL-8 and tumor necrosis factor (TNF)-α [14]. Comparing the cytokine response to infection may provide an important mechanistic insight into any interaction between asthma and COVID-19. In this study, we investigated the role of pre-existing asthma as a comorbidity in disease severity of COVID-19 by comparison of immune response via circulating cytokines.

## Method

### Patients and samples

Plasma samples and clinical information were collected from a total of 109 individuals, including 80 COVID-19 patients [25 mild (CM), 36 severe (CS) and 19 critical (CC)] and 29 healthy volunteers (HV), at the John Radcliffe Hospital, Oxford, UK. Among the COVID-19 patients, 16 patients had pre-existing asthma (3 in CM, 10 in CS, and 3 in CC groups) (Table 1). The diagnosis and severity classification of COVID-19 were in accordance with the guideline by the World Health Organization [21]. The history of asthma comorbidity was defined using hospital episode statistics ICD10 codes, and it was under control on regular inhaled corticosteroids when hospitalized for COVID-19 infection. Ethics were approved by the research ethics committee (REC) at Yorkshire & The Humber—Sheffield (GI Biobank Study 16/YH/0247), and written informed consent was obtained from each patient before sample collection.

**Table 1:** Demographic details of the patients with asthma across different COVID-19 severity groups

	Asthma with critical COVID-19 (n = 3)	Asthma with severe COVID-19 (n = 10)	Asthma with mild COVID-19 (n = 3)
Age	70 ± 11	56 ± 16	37 ± 17
BMI (kg/cm^2^)	27.52 ± 6.74	28.92 ± 4.63	26.94 ± 1.74
Gender (M/F)	3/0	7/3	1/2
Asthma severity (mild/severe)	3/0	10/0	3/0
Eosinophilic asthma	0	2	1
Blood eosinophil counts (cells/μL)	150 ± 36	210 ± 108	220 ± 0
PEF (L/min)	520 ± 0	500 ± 151	150 ± 0
FEV1 (L/min)	NA	2.78 ± 1.28	NA
FEV1/FVC (%)	NA	85.00 ± 4.24	NA
FeNO (ppb)	NA	NA	NA
ICS	Yes	Yes	Yes
OCS	No	No	No
Biological treatments	No	No	No

*BMI* body mass index, *FeNO* fractional exhaled nitric oxide, *FEV1* forced expiratory volume in one second, *FVC* forced vital capacity, *ICS* inhaled corticosteroids, *OCS* oral corticosteroids, *PEF* peak expiratory flow

### Luminex assay

The concentrations of 51 proteins in the plasma samples were measured with Human Magnetic Luminex Kits (Bio-techne) in 3 panels according to the manufacturer’s instructions, including: C–C motif ligand (CCL)2/3/4/11/17/18/19/20, CD40 Ligand (CD40L), CD163, complement component 5a (C5a), C-X-C motif chemokine ligand (CXCL)1/5/10, epidermal growth factor (EGF), basic fibroblast growth factor (FGF2), granulocyte colony-stimulating factor (G-CSF), granulocyte–macrophage colony-stimulating factor (GM-CSF), granzyme B (GrB), interferon (IFN)-α/β/γ, IL-1α/1β/2/3/5/6/8/10/12/13/15/17A/23/33, lactoferrin (LF), Lipocalin-2 (LCN2), Lymphotoxin-alpha (LTα), macrophage colony-stimulating factor (M-CSF), Myeloperoxidase (MPO), beta-nerve growth factor (β-NGF), Oncostatin M (OSM), S100 calcium-binding protein A9 (S100A9), stem cell growth factor (SCGF), tissue factor (TF), tissue factor pathway inhibitor (TFPI), transforming growth factor alpha (TGFα), Thrombopoietin (THPO), TNF, and triggering receptor expressed on myeloid cells 1 (TREM-1). The Results were obtained by the Bio-Rad Bio-Plex® 200 Systems.

### Data analysis

#### Clinical characteristics and plasma proteins

Clinical traits and the selected plasma proteins between COVID-19 patients with and without asthma were compared by unpaired independent Student’s t-test and Chi-square (χ^2^) test or Fisher’s exact test in SPSS.

#### Heatmap

The heatmaps to compare the levels of the 51 proteins were generated with R (ComplexHeatmap) and coloured by the fold-change in log_10_ of the fluorescence intensity (FI) after normalization against the mean value of patients without asthma.

#### Volcano plots

The *p*-values of the 51 proteins in the Volcano plots were obtained from SPSS by using a two-tailed independent student’s t-test on the natural log (Ln) of the FI between patients with and without asthma. The fold changes were calculated by the mean values of Ln(FI) of each protein in asthma against that in non-asthma.

#### Uniform manifold approximation and projection (UMAP)

The UMAPs were calculated using the MATLAB package [22]. The UMAP was partially supervised, with 2/3rds of the patients in each condition randomly chosen to train the network. The UMAP was set to have 45 nearest neighbours, a minimum distance of 0.3 with a correlation metric. The UMAP was reduced over 20,000 epochs.

#### Correlation analysis

The FI of the 51 proteins from 80 COVID-19 patients were used for Pearson correlation analysis to calculate the correlation coefficient (r). Correlation matrix was plotted by R (corrplot) based on the Pearson correlation coefficient and *p*-values. Correlation network was generated by R (ggraph) using those with absolute correlation coefficients of > 0.3. Severity was scored as CM = 1, CS = 2 and CC = 3.

## Results

In total, 16 patients with a pre-existing comorbidity of asthma were found in this cohort, of which 3 in mild, 10 in severe, and 3 in critical COVID-19. The overall prevalence of asthma in COVID-19 was 20.0%. These patients had no significant differences in body mass index (BMI), gender, ethnicity, or smoking status compared with non-asthma patients in the cohort (Table 2).

**Table 2:** Comparison of clinical traits between COVID-19 patients with and without asthma (mean ± SD)

Clinical features	Critical COVID-19						Severe COVID-19			Mild COVID-19		
	Non-Asthma (n = 16)		Asthma (n = 3)		P		Non-Asthma (n = 26)	Asthma (n = 10)	P	Non-Asthma (n = 22)	Asthma (n = 3)	P
Age	55 ± 12		70 ± 11		0.077		67 ± 16	56 ± 16	0.066	70 ± 20	37 ± 17	0.014
BMI (kg/cm^2^)	29.57 ± 7.10		27.52 ± 6.74		0.741		31.45 ± 6.51	28.92 ± 4.63	0.475	24.92 ± 3.50	26.94 ± 1.74	0.465
Gender (M/F)	11/5		3/0		0.530		13/13	7/3	0.456	10/12	1/2	1.000
Ethnicity					0.308				0.622			0.071
Caucasians	6 (40.0%)		3 (100.0%)				19 (76.0%)	9 (90.0%)		15 (78.9%)	2 (66.7%)	
Asian	3 (20.0%)		0 (0%)				1 (4.0%)	1 (10.0%)		3 (15.8%)	0 (0%)	
African	0 (0%)		0 (0%)				3 (12.0%)	0 (0%)		0 (0%)	1 (33.3%)	
Mediterranean	0 (0%)		0 (0%)				0 (0%)	0 (0%)		0 (0%)	0 (0%)	
Mixed	1 (6.7%)		0 (0%)				1 (4.0%)	0 (0%)		0 (0%)	0 (0%)	
Others	5 (33.3%)		0 (0%)				1 (4.0%)	0 (0%)		1 (5.3%)	0 (0%)	
Smoking					1.000				0.304			0.242
Non-smokers	10 (66.7%)		2 (66.7%)				17 (68.0%)	4 (44.4%)		9 (52.9%)	3 (100.0%)	
Ex-smokers	5 (33.3%)		1 (33.3%)				7 (28.0%)	5 (55.6%)		8 (47.1%)	0 (0%)	
Active smokers	0 (0%)		0 (0%)				1 (4.0%)	0 (0%)		0 (0%)	0 (0%)	
Days of symptoms to sampling	12.81 ± 5.41		10.33 ± 3.22		0.459		9.31 ± 5.13	11.60 ± 5.36	0.243	6.86 ± 3.58	13.33 ± 11.15	0.037
Comorbidities												
CCI		1.44 ± 4.07		6.67 ± 2.52		0.049	8.77 ± 10.11	7.60 ± 9.36	0.753	7.32 ± 9.44	4.00 ± 0	0.556
Hypertension		6 (37.5%)		2 (66.7%)		0.546	14 (53.8%)	3 (30.0%)	0.274	8 (36.4%)	0 (0%)	0.527
CRD		1 (6.3%)		0 (0.0%)		1.000	6 (23.1%)	1 (10.0%)	0.645	–	–	–
CCD		1 (6.3%)		1 (33.3%)		0.298	3 (11.5%)	1 (10.0%)	1.000	5 (22.7%)	0 (0%)	1.000
DM		3 (18.8%)		1 (33.3%)		0.530	15 (57.7%)	3 (30.0%)	0.137	6 (27.3%)	0 (0%)	0.554
Haemcancer		–		–		–	–	–	–	–	–	–
Other cancer		–		–		–	–	–	–	1 (4.5%)	0 (0%)	1.000
Liver diseases		1 (6.3%)		0 (0%)		1.000	0 (0%)	1 (10.0%)	0.278	–	–	–
Neurological diseases		–		–		–	0 (0%)	1 (10.0%)	0.278	–	–	–
Kidney diseases		–		–		–	–	–	–	1 (4.5%)	0 (0%)	1.000
Transplantation		–		–		–	–	–	–	–	–	–
Rheumatological diseases		–		–		–	–	–	–	1 (4.5%)	0 (0%)	1.000
Immunosuppression		–		–		–	–	–	–	0 (0%)	1 (33.3%)	0.120
Stroke/dementia		–		–		–	–	–	–	2 (9.1%)	0 (0%)	1.000
SaO_2_/FiO_2_		1.72 ± 0.49		1.83 ± 0.57		0.783	2.65 ± 1.10	2.75 ± 1.30	0.845	4.57 ± 0.10	4.52 ± 0.13	0.569
WBC (×10^9^/L)		9.45 ± 3.54		7.74 ± 2.52		0.441	7.85 ± 3.59	7.54 ± 2.68	0.814	7.58 ± 2.81	5.48 ± 4.32	0.273
Neutrophils (×10^9^/L)		8.06 ± 3.44		6.89 ± 2.41		0.585	6.23 ± 3.48	5.76 ± 2.89	0.723	5.66 ± 2.50	3.67 ± 3.52	0.235
Eosinophils (×10^9^/L)		0.08 ± 0.08		0.08 ± 0.09		0.909	0.03 ± 0.04	0.09 ± 0.10	0.059	0.06 ± 0.06	0.07 ± 0.06	0.711
Lymphocytes (×10^9^/L)		0.93 ± 0.37		0.49 ± 0.15		0.062	1.01 ± 0.43	1.16 ± 0.40	0.387	1.35 ± 0.57	1.12 ± 0.22	0.492
Monocytes (×10^9^/L)		0.39 ± 0.21		0.26 ± 0.21		0.325	0.55 ± 0.32	0.52 ± 0.31	0.761	0.50 ± 0.26	0.63 ± 0.66	0.545
Haemoglobin (g/L)		114.31 ± 22.53		111.33 ± 15.89		0.831	126.09 ± 15.96	126.88 ± 15.01	0.905	126.85 ± 16.54	127.67 ± 6.03	0.935
Platelets (×10^9^/L)		265.31 ± 111.65		234.33 ± 39.51		0.647	292.16 ± 121.19	356.50 ± 90.69	0.163	272.68 ± 98.73	259.67 ± 34.15	0.826
Lactate (mmol/L) (highest)		1.294 ± 0.6567		1.100 ± 0.1000		0.625	1.529 ± 0.4327	0.962 ± 0.2560	0.002	1.520 ± 0.3824	0.900 ± 0.1414	0.053
Bicarbonate (mmol/L) (highest)		24.71 ± 4.04		25.77 ± 2.66		0.673	24.37 ± 3.30	25.64 ± 1.56	0.316	24.88 ± 2.73	23.95 ± 1.48	0.658
CRP (highest)		233.90 ± 156.36		207.93 ± 60.54		0.784	163.73 ± 85.65	106.82 ± 49.61	0.073	74.87 ± 61.52	56.43 ± 30.81	0.622
ALP (highest)		97.81 ± 45.51		69.33 ± 13.58		0.307	103.71 ± 66.26	106.50 ± 93.33	0.929	118.06 ± 92.09	64.00 ± 8.19	0.335
ALT (highest)		66.25 ± 42.61		55.33 ± 17.10		0.673	50.71 ± 42.91	74.89 ± 86.65	0.311	55.56 ± 79.51	32.00 ± 22.11	0.624
d-Dimer (timepoint)		13,585.44 ± 22,511.816		2240.00 ± 0		0.645	2574.86 ± 6268.18	3491.00 ± 5068.18	0.730	9842.67 ± 26,319.981	2738.00 ± 2586.597	0.723
LDH (timepoint)		600.00 ± 79.076		–		–	526.00 ± 281.27	315.50 ± 102.19	0.178	433.67 ± 104.45	–	–
CK (timepoint)		299.00 ± 450.172		–		–	332.64 ± 849.60	116.20 ± 89.85	0.586	211.90 ± 180.90	–	–
OS score		0.76 ± 1.19		1.96 ± 0.96		0.122	1.95 ± 1.47	1.04 ± 1.98	0.138	1.81 ± 1.77	− 1.13 ± 1.11	0.011
Vasopressors		14 (87.5%)		3 (100.0%)		1.000	2 (7.7%)	2 (20.0%)	0.305	–	–	–
Days on vasopressors		10.13 ± 8.84		8.00 ± 5.57		0.697	1.25 ± 3.15	2.50 ± 4.36	0.579	–	–	–
RRT		6 (37.5%)		0 (0%)		0.517	1 (3.8%)	1 (10.0%)	0.484	–	–	–
Days on RRT		3.019 ± 5.73		–		–	0.88 ± 2.48	0.50 ± 1.00	0.781	–	–	–
Days on ventilation (days)		24.44 ± 11.88		11.67 ± 4.16		0.089	9.13 ± 12.08	7.00 ± 8.45	0.761	–	–	–
Hospital LOS (days)		40.44 ± 20.36		17.67 ± 0.58		0.076	14.42 ± 14.34	10.70 ± 6.78	0.439	7.14 ± 6.72	4.67 ± 3.79	0.544
Mortality		2 (12.5%)		1 (33.3%)		0.422	7 (26.9%)	1 (10.0%)	0.397	2 (9.1%)	0 (0%)	1.000

*ALP* alkaline phosphatase, *ALT* alanine transaminase, *BMI* body mass index, *CCD* congestive cardiac disease, *CCI* Charlson comorbidity index, *CK* Creatine kinase, *CRD* chronic renal disease, *CRP* C-reactive protein, *DM* diabetes mellitus, *LDH* lactate dehydrogenase, *LOS* length of stay, *NIV* non-invasive ventilation, *NRM* non-rebreather mask, *OS score* OpenSAFELY score, *RA* room air, *RRT* renal replacement therapy, *SFM* simple face mask, *SaO*_*2*_*/FiO*_*2*_ ratio of arterial oxygen saturation to fraction of inspired oxygen, *WBC* white blood cells

### Circulating cytokines/mediators between COVID-19 patients with and without asthma

**Fig. 1:**
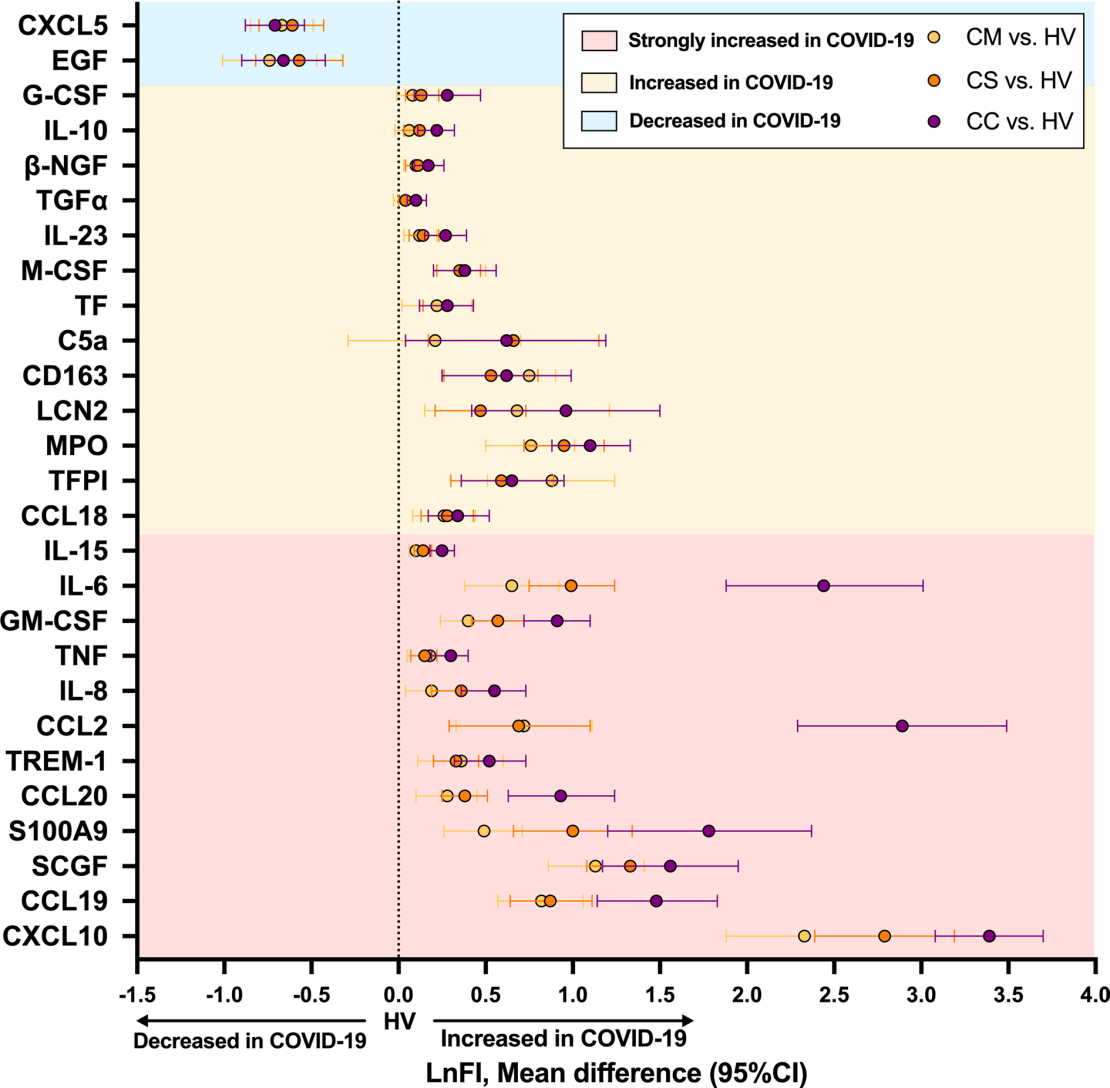
Plasma cytokine enrichment profile in COVID-19. Comparison of mean fluorescence intensity (FI) difference of plasma proteins between different disease severities of COVID-19 (mild-CM, severe-CS, and critical-CC) and healthy volunteer (HV) measured with Luminex (95% confidence interval). The different colour-shaded backgrounds indicate the changing direction of the protein levels compared to HV

To understand the impact of asthma comorbidity on the immune response of COVID-19 infection, we investigated the effect of asthma comorbidity on the COVID-19 cytokine profile. A cytokine storm was clearly detected in this COVID-19 cohort. Of the tested 51 plasma proteins, the levels of 25 proteins: CCL2/18/19/20, CD163, C5a, CXCL10, G-CSF, GM-CSF, IL-6/8/10/15/23, LCN2, M-CSF, MPO, β-NGF, S100A9, SCGF, TF, TFPI, TGF-α, TNF and TREM-1, were significantly elevated in the plasma from patients with COVID-19 compared with that from healthy donors (Fig. 1). Among these enriched proteins, 12 exhibited a particularly strong increase in every critical patient: CCL2/19/20, CXCL10, GM-CSF, IL-6/8/15, S100A9, SCGF, TNF and TREM-1. Two cytokines (CXCL5 and EGF) were down-regulated after COVID-19 infection.

**Fig. 2:**
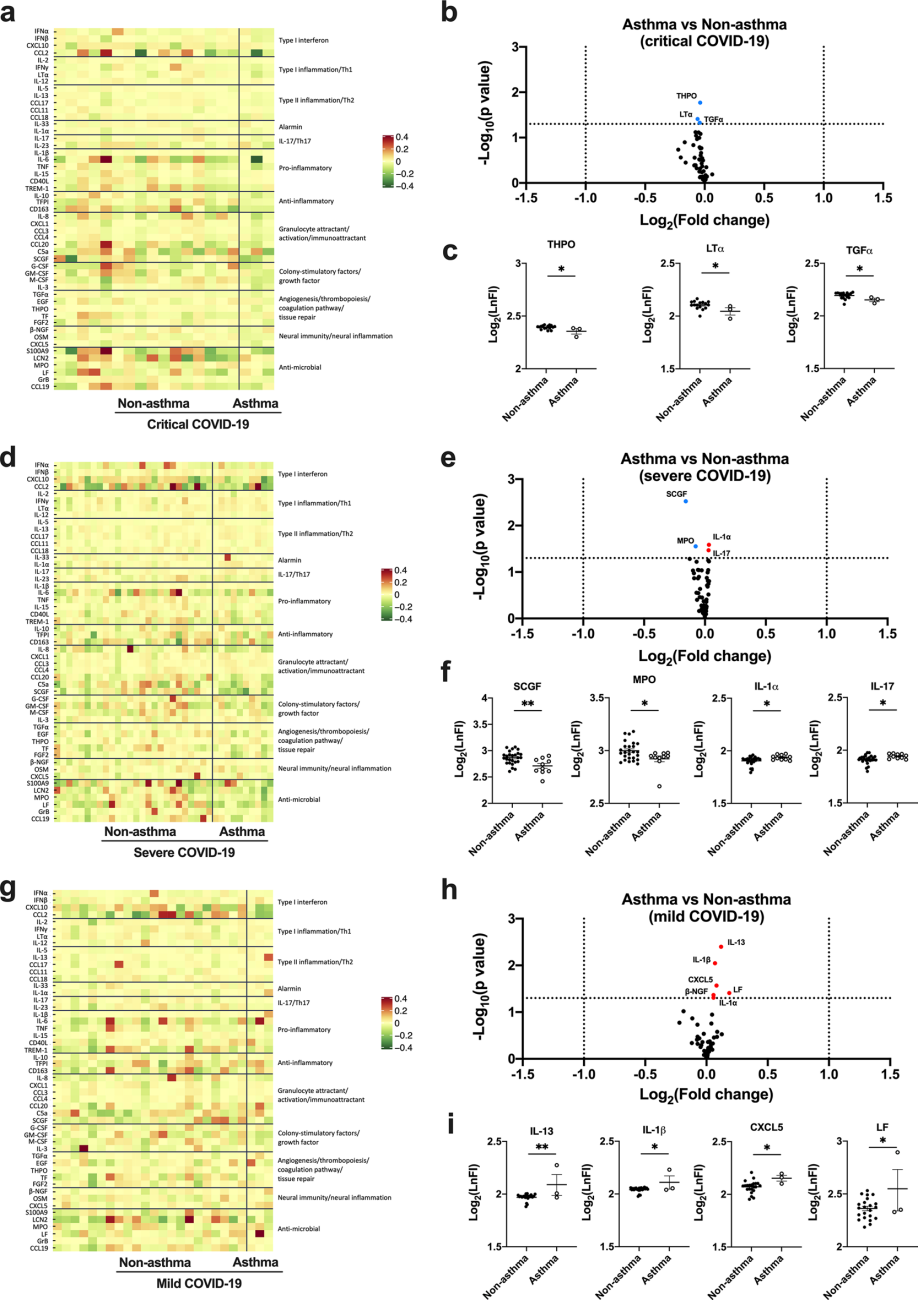
Comparison of plasma cytokine profiles between COVID-19 patients with and without asthma within different severity groups. **a**, **d**, **g** Heatmaps showing the comparison of FI levels of 51 plasma proteins between asthmatic and non-asthmatic patients in critical (**a**), severe (**d**), and mild (**g**) COVID-19 groups detected with Luminex assay. **b**, **e**, **h** Volcano plots showing differential abundance of 51 plasma proteins between asthmatic and non-asthmatic patients in critical (**b**), severe (**e**), and mild (**h**) COVID-19 groups. The horizontal dash-lines indicate p-value of 0.05. The proteins with significantly lower levels in asthma group were shaded in blue, and significantly higher were shaded in red. **c**, **f**, **i** Scatterplots of FI levels of cytokines showed significant difference between asthmatic and non-asthmatic patients in critical (**c**), severe (**f**), and mild (**i**) COVID-19 groups. *p < 0.05 and **p < 0.001

**Fig. 3:**
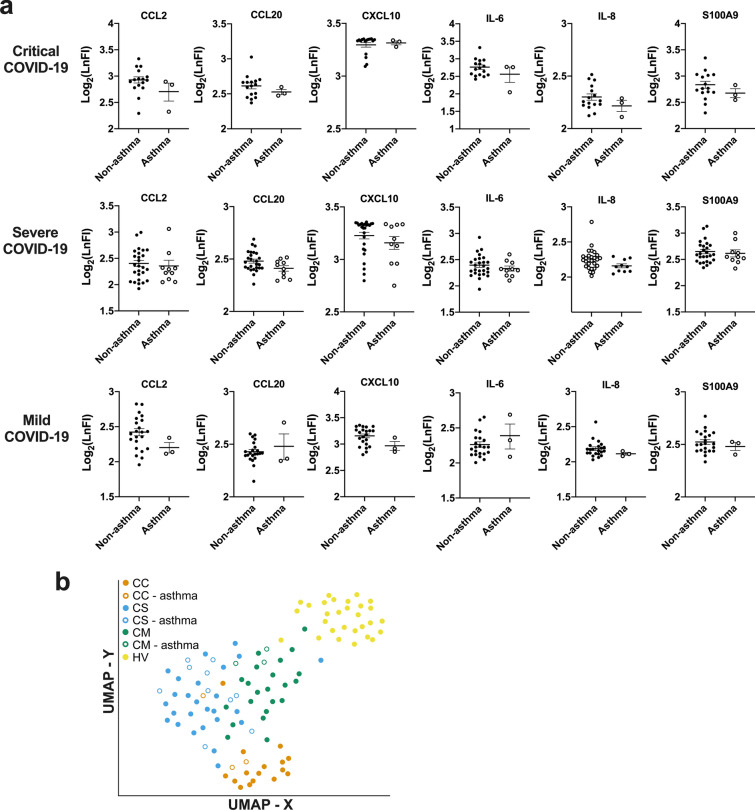
Comparison of plasma cytokine levels between COVID-19 patients with and without asthma. **a** Scatterplots of FI levels of CCL2, CCL20, CXCL10, IL-6, IL-8 and S100A9 obtained from Luminex assay in plasma from patients with and without asthma in different COVID-19 severity groups. **b** UMAP analysis showing the distribution of asthmatic patients (hollow circle) in the clusters of different COVID-19 severity groups (shaded with colours) based on their plasma protein profiles. HV was used as a control group

The levels of these enriched plasma proteins were compared between asthmatic and non-asthmatic patients within same COVID-19 severity groups (Fig. 2). In general, no obvious differences were observed between the two populations. In the critical COVID-19 group, the heatmap (Fig. 2a) and volcano plot (Fig. 2b) even showed that the levels of some circulating cytokines were marginally lower in patients with asthma than those without asthma, particularly on THPO, LTα, and TGFα (Fig. 2c). In severe COVID-19, a similar pattern was observed (Fig. 2d, e) but weakly lower on SCGF and MPO and weakly higher on IL-1α and IL-17 (Fig. 2f). In mild COVID-19, the cytokines weakly elevated in patients with asthma were IL-1β, IL-13, CXCL5, and LF (Fig. 2g–i). However, in all cases, the cytokines that were weakly upregulated in the asthma group were not those upregulated in COVID-19 cytokine storm conditions. In contrary, some important cytokines in COVID-19 cytokine storm such as CXCL10, IL-6, CCL2, CCL20, IL-8 and S100A9 showed a trend of slightly lower levels in patients with asthma in all three COVID-19 severity groups (Fig. 3a). UMAP also confirmed that asthmatic patients in different COVID-19 severity groups are randomly distributed in the same severity clusters, and have no obvious difference on the cytokine profiles from non-asthmatic patients (Fig. 3b).

### Correlation between asthma and clinical outcomes and circulating proteins in patients with COVID-19

**Fig. 4:**
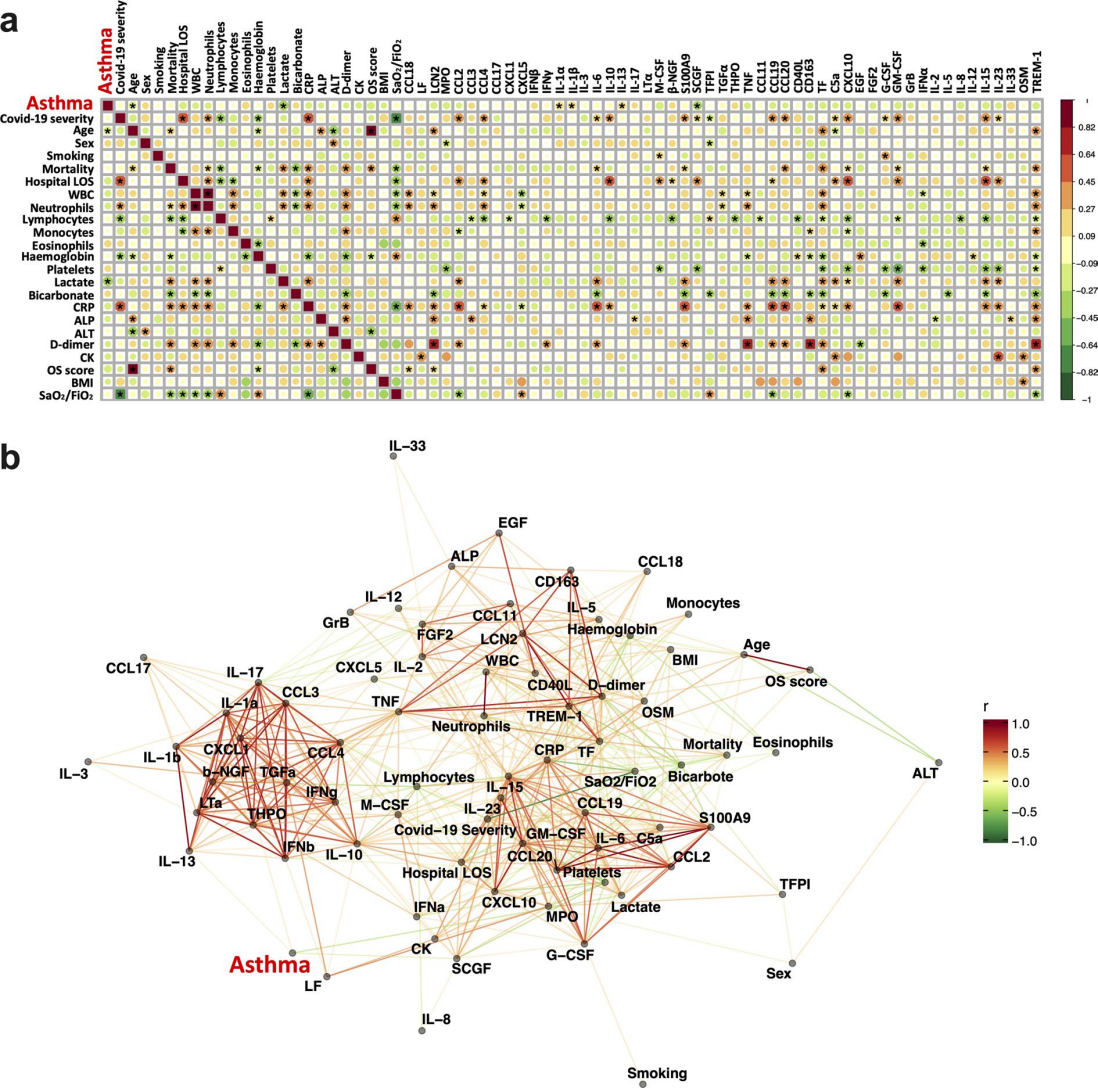
Correlation analysis between asthma and clinical traits/cytokines in COVID-19 patients. **a** Correlation of asthma and clinical traits/circulating proteins in patients with COVID-19. The size of the dots represents the significance level of the correlation, with a “*” indicating *p* < 0.05. Correlation coefficients (r) are indicated by colour. **b** Network of asthma and clinical traits/circulating proteins correlations in patients with COVID-19. The colours of the lines indicate the correlation coefficients (r)

To further explore the role of asthma on COVID-19 severity, mortality, and the circulating cytokine storm, a correlation analysis was conducted. It showed that asthma was not associated with severity scores of COVID-19 or the relevant clinical outcomes such as mortality and hospital length of stay (Fig. 4a). In addition, we found that higher blood lactate level was associated with higher mortality and that higher blood SCGF level was associated with severity of COVID-19, however, asthma was negatively correlated with these two covariates. Furthermore, asthma was not associated with any of the clinical traits or circulating cytokines that were significantly positive-correlated [hospital length of stay (LOS), blood neutrophil counts, C-reactive protein (CRP), CCL2, CCL4, IL-6, IL-10, S100A9, SCGF, CCL19, CCL20, C5a, CXCL10, G-CSF, GM-CSF, IL-15, IL-23] or negative-correlated [lymphocyte counts, haemoglobin, the ratio of arterial oxygen saturation to fraction of inspired oxygen (SaO_2_/FiO_2_) and TFPI] with COVID-19 severity scores. Similarly, asthma was also not associated with the clinical traits or cytokines that were positively [blood neutrophil counts, lactate, CRP, d-dimer, OpenSAFELY (OS) score, IL-6, S100A9, CCL20, TF, CXCL10, GM-CSF, IL-15, TREM-1] or negatively (blood lymphocyte counts, haemoglobin, bicarbonate and SaO_2_/FiO_2_) correlated with mortality. The correlation network analysis also did not show strong linkage between asthma and COVID-19 disease severity and mortality (Fig. 4b).

### Clinical characteristics between COVID-19 patients with and without asthma

To confirm the above observation, the clinical traits between patients with and without asthma in different COVID-19 severity groups were compared (Table 2). Similar burden of comorbidities, oxygen level (SaO_2_/FiO_2_), neutrophil counts, platelet counts, lactate, and CRP were detected in both groups. As for the outcomes, we also did not observe any significant differences on the use of and the duration on vasopressors, renal replacement therapy, or ventilation. The overall mortality in COVID-19 patients with asthma (12.5%) was not higher than that in patients without asthma (17.2%) in total cohort, nor was in the different COVID-19 severity groups.

Furthermore, although the age distribution showed a slight increase with severity of COVID-19 in the patients with asthma (37 ± 17 in mild, 56 ± 16 in severe and 70 ± 11 in critical) than that in the patients without asthma (70 ± 20 in mild, 67 ± 16 in severe and 55 ± 12 in critical), the prevalence of asthma in COVID-19 severity groups (12.0% in mild, 27.8% in severe and 15.8% in critical) did not exhibit a clear correlation between asthma and the severity of COVID-19.

## Discussion

Although different prevalences of asthma in patients with COVID-19 have been reported, a consistent finding is that this prevalence is lower than that in the general populations without COVID-19 [23]. In China, chronic respiratory diseases including asthma have been reported in 2.4% of 44,672 confirmed cases [24] and in the epicentre of Wuhan this figure was only 0.9% [11], which were lower than the reported asthma prevalence in the nationwide (4.2%) [25] and in Wuhan (6.4%) [26]. In the UK, pre-existing asthma has been identified in 14.5% of 20,133 hospitalised patients with COVID-19 [13], while the nationwide prevalence of asthma was 18.15% in the whole population [27]. Furthermore, no difference was found in the prevalence of asthma between severe (1.1%) and non-severe (0.7%) COVID-19 in a previous study [11]. It has also been reported that the percentage of severe COVID-19 cases in asthmatic patients (4.6%) was significantly lower than that in non-asthmatic patients (25.0%) [14]. In this Oxford cohort, a similar pattern was observed that the prevalence of asthma in COVID-19 patients was not significantly higher than that in general population, and there was no difference of prevalence of pre-existing asthma among different COVID-19 severities. Although we found more severe and critical COVID-19 cases in patients with asthma than that reported from China, no significant difference was shown compared to that in patients without asthma. Therefore, our results support the emerging idea that patients with underlying comorbidity of asthma are not at higher risk of severe COVID-19 infection [9, 28]. However, it is still unclear whether SARS-CoV-2 would increase asthma exacerbation although viral infection is recognized as one of the risk factors for asthma exacerbation, therefore, further investigations on this aspect are needed.

Cytokine storm is one of the key features in COVID-19 infection. Many cytokines such as CXCL10, IL-6, CCL2, IL-8, MPO have been reported to be associated with disease severity and mortality [5, 20, 29]. In order to investigate any potential impact of pre-existing asthma as a comorbidity on the immunological mechanism of COVID-19 disease, we measured and compared the circulating proteins in plasma which covered a spectrum of 51 proteins. Our data demonstrated strong enrichment of CCL2/19/20, CXCL10, GM-CSF, IL-6/8/15, S100A9, SCGF, TNF and TREM-1 in COVID-19, which could potentially play a critical role on the pathogenesis of the disease. No further enhancement of these enrichments was detected in COVID-19 patients with asthma. By contrast, we detected a few cytokines (CXCL5, IL-1α/1β/13/17, LF) weakly elevated in asthma groups, which are not relevant to pathogenesis of COVID-19 as the levels of these proteins are not changed or even decreased in general COVID-19 patients. Some of these observations were consistent with the findings in the study by Song et al*.* [14]. Therefore, asthma comorbidity does not appear to modify the cytokine storm cascade after COVID-19 infection, and any difference in disease severity is unlikely to be explained on this basis.

The influences of pre-existing asthma on the outcomes of COVID-19 are still unknown. Although a recent post hoc analysis (OpenSAFELY study) reported an association between uncontrolled asthma and COVID-19-related death, the population they analysed were not only patients with COVID-19 disease [19], therefore, it is still unclear whether COVID-19 patient with pre-existing asthma would end up with worse outcomes compared to those without asthma. So far, there is only one study that reports the effect of asthma as well as chronic obstructive pulmonary disease (COPD) on the outcomes of patients with COVID-19 [14]. In that study, no significant difference was found in mortality between COVID-19 patients with and without asthma, but patients with COPD had significantly increased mortality [14]. In our study, we not only compared mortality but also other clinical traits between patients with and without asthma in different COVID-19 disease severity groups. No correlation between asthma and COVID-19-related clinical features was detected in our cohort. Hence, our data suggests that pre-existing asthma is not associated with worse clinical outcomes of COVID-19. Of course, future bigger COVID-19 cohort investigation including different asthma phenotypes would be useful to further improve our understanding of the effect of asthma in COVID-19.

## Conclusions

Based on this cohort study, pre-existing asthma was not associated with an enhanced cytokine storm after COVID-19 infection, and did not have strong effects on COVID-19 progression and mortality.

## Data Availability

The datasets used and/or analysed during the current study are available from the corresponding author on reasonable request.

## Abbreviations

ACE2: Angiotension-converting enzyme 2
BMI: Body mass index
β-NGF: Beta-nerve growth factor
C5a: Complement component 5a
CC: Critical COVID-19
CCL: C–C motif ligand
CM: Mild COVID-19
CRP: C-reactive protein
CS: Severe COVID-19
CXCL: C-X-C motif chemokine ligand
EGF: Epidermal growth factor
G-CSF: Granulocyte colony-stimulating factor
GM-CSF: Granulocyte-macrophage colony-stimulating factor
FI: Fluorescence intensity
HV: Healthy volunteer
IL: Interleukin
LF: Lactoferrin
MPO: Myeloperoxidase
S100A9: S100 calcium-binding protein A9
SaO_2_/FiO_2_: Ratio of arterial oxygen saturation to fraction of inspired oxygen
SCGF: Stem cell growth factor
TF: Tissue factor
TFPI: Tissue factor pathway inhibitor
TGFα: Transforming growth factor alpha
TNF: Tumor necrosis factor
TREM-1: Triggering receptor expressed on myeloid cells 1
UMAP: Uniform manifold approximation and projection

## References

[1] Zhu N, Zhang D, Wang W, Li X, Yang B, Song J, Zhao X, Huang B, Shi W, Lu R, Niu P, Zhan F, Ma X, Wang D, Xu W, Wu G, Gao GF, Tan W, China Novel Coronavirus Investigating and Research Team (2020). A novel coronavirus from patients with pneumonia in China, 2019. N Engl J Med.

[2] Zhou P, Yang XL, Wang XG, Hu B, Zhang L, Zhang W, Si HR, Zhu Y, Li B, Huang CL, Chen HD, Chen J, Luo Y, Guo H, Jiang RD, Liu MQ, Chen Y, Shen XR, Wang X, Zheng XS, Zhao K, Chen QJ, Deng F, Liu LL, Yan B, Zhan FX, Wang YY, Xiao GF, Shi ZL (2020). A pneumonia outbreak associated with a new coronavirus of probable bat origin. Nature.

[3] Wang Q, Zhang Y, Wu L, Niu S, Song C, Zhang Z, Lu G, Qiao C, Hu Y, Yuen KY, Wang Q, Zhou H, Yan J, Qi J (2020). Structural and functional basis of SARS-CoV-2 entry by using human ACE2. Cell.

[4] Menter T, Haslbauer JD, Nienhold R, Savic S, Hopfer H, Deigendesch N, Frank S, Turek D, Willi N, Pargger H, Bassetti S, Leuppi JD, Cathomas G, Tolnay M, Mertz KD, Tzankov A (2020). Postmortem examination of COVID-19 patients reveals diffuse alveolar damage with severe capillary congestion and variegated findings in lungs and other organs suggesting vascular dysfunction. Histopathology.

[5] Huang C, Wang Y, Li X, Ren L, Zhao J, Hu Y, Zhang L, Fan G, Xu J, Gu X, Cheng Z, Yu T, Xia J, Wei Y, Wu W, Xie X, Yin W, Li H, Liu M, Xiao Y, Gao H, Guo L, Xie J, Wang G, Jiang R, Gao Z, Jin Q, Wang J, Cao B (2020). Clinical features of patients infected with 2019 novel coronavirus in Wuhan, China. Lancet.

[6] Pavord ID, Beasley R, Agusti A, Anderson GP, Bel E, Brusselle G, Cullinan P, Custovic A, Ducharme FM, Fahy JV, Frey U, Gibson P, Heaney LG, Holt PG, Humbert M, Lloyd CM, Marks G, Martinez FD, Sly PD, von Mutius E, Wenzel S, Zar HJ, Bush A (2018). After asthma: redefining airways diseases. Lancet.

[7] Zheng XY, Xu YJ, Guan WJ, Lin LF (2018). Regional, age and respiratory-secretion-specific prevalence of respiratory viruses associated with asthma exacerbation: a literature review. Arch Virol.

[8] Riggioni C, Comberiati P, Giovannini M, Agache I, Akdis M, Alves-Correia M, Antó JM, Arcolaci A, Azkur AK, Azkur D, Beken B, Boccabella C, Bousquet J, Breiteneder H, Carvalho D, De Las VL, Diamant Z, Eguiluz-Gracia I, Eiwegger T, Eyerich S, Fokkens W, Gao YD, Hannachi F, Johnston SL, Jutel M, Karavelia A, Klimek L, Moya B, Nadeau KC, O'Hehir R, O'Mahony L, Pfaar O, Sanak M, Schwarze J, Sokolowska M, Torres MJ, van de Veen W, van Zelm MC, Wang Y, Zhang L, Jiménez-Saiz R, Akdis CA (2020). A compendium answering 150 questions on COVID-19 and SARS-CoV-2. Allergy.

[9] Carli G, Cecchi L, Stebbing J, Parronchi P, Farsi A (2020). Is asthma protective against COVID-19?. Allergy.

[10] Johnston SL (2020). Asthma and COVID-19: is asthma a risk factor for severe outcomes?. Allergy.

[11] Li X, Xu S, Yu M, Wang K, Tao Y, Zhou Y, Shi J, Zhou M, Wu B, Yang Z, Zhang C, Yue J, Zhang Z, Renz H, Liu X, Xie J, Xie M, Zhao J (2020). Risk factors for severity and mortality in adult COVID-19 inpatients in Wuhan. J Allergy Clin Immunol.

[12] Grasselli G, Zangrillo A, Zanella A, Antonelli M, Cabrini L, Castelli A, Cereda D, Coluccello A, Foti G, Fumagalli R, Iotti G, Latronico N, Lorini L, Merler S, Natalini G, Piatti A, Ranieri MV, Scandroglio AM, Storti E, Cecconi M, Pesenti A, COVID-19 Lombardy ICU Network (2020). Baseline characteristics and outcomes of 1591 patients infected with SARS-CoV-2 admitted to ICUs of the Lombardy Region, Italy. JAMA.

[13] Docherty AB, Harrison EM, Green CA, Hardwick HE, Pius R, Norman L, Holden KA, Read JM, Dondelinger F, Carson G, Merson L, Lee J, Plotkin D, Sigfrid L, Halpin S, Jackson C, Gamble C, Horby PW, Nguyen-Van-Tam JS, Ho A, Russell CD, Dunning J, Openshaw PJ, Baillie JK, Semple MG, ISARIC4C investigators (2020). Features of 20 133 UK patients in hospital with covid-19 using the ISARIC WHO clinical characterisation protocol: prospective observational cohort study. BMJ.

[14] Song J, Zeng M, Wang H, Qin C, Hou HY, Sun ZY, Xu SP, Wang GP, Guo CL, Deng YK, Wang ZC, Ma J, Pan L, Liao B, Du ZH, Feng QM, Liu Y, Xie JG, Liu Z (2020). Distinct effects of asthma and COPD comorbidity on disease expression and outcome in patients with COVID-19. Allergy.

[15] Jackson DJ, Busse WW, Bacharier LB, Kattan M, O'Connor GT, Wood RA, Visness CM, Durham SR, Larson D, Esnault S, Ober C, Gergen PJ, Becker P, Togias A, Gern JE, Altman MC (2020). Association of respiratory allergy, asthma, and expression of the SARS-CoV-2 receptor ACE2. J Allergy Clin Immunol.

[16] Kimura H, Francisco D, Conway M, Martinez FD, Vercelli D, Polverino F, Billheimer D, Kraft M (2020). Type 2 inflammation modulates ACE2 and TMPRSS2 in airway epithelial cells. J Allergy Clin Immunol.

[17] Camiolo M, Gauthier M, Kaminski N, Ray A, Wenzel SE (2020). Expression of SARS-CoV-2 receptor ACE2 and coincident host response signature varies by asthma inflammatory phenotype. J Allergy Clin Immunol.

[18] Zhou F, Yu T, Du R, Fan G, Liu Y, Liu Z, Xiang J, Wang Y, Song B, Gu X, Guan L, Wei Y, Li H, Wu X, Xu J, Tu S, Zhang Y, Chen H, Cao B (2020). Clinical course and risk factors for mortality of adult inpatients with COVID-19 in Wuhan, China: a retrospective cohort study. Lancet.

[19] Williamson EJ, Walker AJ, Bhaskaran K, Bacon S, Bates C, Morton CE, Curtis HJ, Mehrkar A, Evans D, Inglesby P, Cockburn J, McDonald HI, MacKenna B, Tomlinson L, Douglas IJ, Rentsch CT, Mathur R, Wong AYS, Grieve R, Harrison D, Forbes H, Schultze A, Croker R, Parry J, Hester F, Harper S, Perera R, Evans SJW, Smeeth L, Goldacre B (2020). Factors associated with COVID-19-related death using OpenSAFELY. Nature.

[20] Chen G, Wu D, Guo W, Cao Y, Huang D, Wang H, Wang T, Zhang X, Chen H, Yu H, Zhang X, Zhang M, Wu S, Song J, Chen T, Han M, Li S, Luo X, Zhao J, Ning Q (2020). Clinical and immunological features of severe and moderate coronavirus disease 2019. J Clin Invest.

[21] World Health Organization, Clinical management of COVID-19. Interim guidance. https://www.who.int/publications/i/item/clinical-management-of-covid-19. Accessed 27 May 2020.

[22] MATLAB Central File Exchange. Uniform manifold approximation and projection (UMAP), 2020. https://uk.mathworks.com/matlabcentral/fileexchange/71902-uniform-manifold-approximation-and-projection-umap. Accessed 16 Dec 2020.

[23] Halpin DMG, Faner R, Sibila O, Badia JR, Agusti A (2020). Do chronic respiratory diseases or their treatment affect the risk of SARS-CoV-2 infection?. Lancet Respir Med.

[24] Epidemiology Working Group For NCIP Epidemic Response, Chinese Center for Disease Control and Prevention (2020). The epidemiological characteristics of an outbreak of 2019 novel coronavirus diseases (COVID-19) in China. Zhonghua Liu Xing Bing Xue Za Zhi.

[25] Huang K, Yang T, Xu J, Yang L, Zhao J, Zhang X, Bai C, Kang J, Ran P, Shen H, Wen F, Chen Y, Sun T, Shan G, Lin Y, Xu G, Wu S, Wang C, Wang R, Shi Z, Xu Y, Ye X, Song Y, Wang Q, Zhou Y, Li W, Ding L, Wan C, Yao W, Guo Y, Xiao F, Lu Y, Peng X, Zhang B, Xiao D, Wang Z, Chen Z, Bu X, Zhang H, Zhang X, An L, Zhang S, Zhu J, Cao Z, Zhan Q, Yang Y, Liang L, Tong X, Dai H, Cao B, Wu T, Chung KF, He J, Wang C, China Pulmonary Health (CPH) Study Group (2019). Prevalence, risk factors, and management of asthma in China: a national cross-sectional study. Lancet.

[26] Wang XD, Zheng M, Lou HF, Wang CS, Zhang Y, Bo MY, Ge SQ, Zhang N, Zhang L, Bachert C (2016). An increased prevalence of self-reported allergic rhinitis in major Chinese cities from 2005 to 2011. Allergy.

[27] To T, Stanojevic S, Moores G, Gershon AS, Bateman ED, Cruz AA, Boulet LP (2012). Global asthma prevalence in adults: findings from the cross-sectional world health survey. BMC Public Health.

[28] Avdeev S, Moiseev S, Brovko M, Yavorovskiy A, Umbetova K, Akulkina L, Tsareva N, Merzhoeva Z, Gainitdinova V, Fomin V (2020). Low prevalence of bronchial asthma and chronic obstructive lung disease among intensive care unit patients with COVID-19. Allergy.

[29] Middleton EA, He XY, Denorme F, Campbell RA, Ng D, Salvatore SP, Mostyka M, Baxter-Stoltzfus A, Borczuk AC, Loda M, Cody MJ, Manne BK, Portier I, Harris ES, Petrey AC, Beswick EJ, Caulin AF, Iovino A, Abegglen LM, Weyrich AS, Rondina MT, Egeblad M, Schiffman JD, Yost CC (2020). Neutrophil extracellular traps contribute to immunothrombosis in COVID-19 acute respiratory distress syndrome. Blood.

